# Impacts of Ethanol Production and Drying Conditions on the Chemical, Physical, and Flowability Properties of Distillers Dried Grains With Solubles

**DOI:** 10.3389/fbioe.2021.716634

**Published:** 2021-08-27

**Authors:** Kurt A. Rosentrater, Yanhong Zhang, Brian Wrenn

**Affiliations:** ^1^ Department of Agricultural and Biosystems Engineering, Iowa State University, Ames, IA, United States; ^2^ National Corn to Ethanol Research Center, Edwardsville, IL, United States

**Keywords:** corn, DDGS, distillers grains, drying, fermentation, flowability, physical properties, processing

## Abstract

The production of corn-based ethanol in the U.S. has dramatically increasied in recent years, and consequently so has the quantity of coproduct feed ingredients generated from this segment of the grain processing industry. These streams are almost exclusively utilized as livestock feed, which partially offsets the need for corn in feed rations, but other value-added applications do exist. Because of its use as an animal feed, considerable research has been conducted into the nutritional properties, but to a lesser extent the physical and flowability properties of commercially-produced distillers dried grains with solubles (DDGS). There can be occasions when the quality of coproducts is not consistent. Thus questions regarding the influence of processing operations on the resulting coproduct characteristics must be examined. The objective of this research was to conduct extensive physical and flowability property analyses on DDGS samples which were produced under varying conditions in a pilot plant-scale ethanol plant, in order to investigate the effects of various manufacturing operations (specifically ethanol production and drying conditions) on the resulting properties of the DDGS. Using various laboratory methods, a variety of properties, including bulk density and angle of repose, were determined. DDGS fat content was highly correlated with aerated and packed bulk densities, which indicates that fat level plays a key role in flowability behavior. Future studies should examine this potential relationship in more depth, especially as the industry has moved to fat reduction via oil separation processes.

## Introduction

Distillers dried grains with solubles (DDGS) is one of the key coproducts of corn based dry grind fuel ethanol production. An exponential increase in the ethanol industry, and thus DDGS, has occurred over the last several years. During ethanol production, each bushel of corn (∼56 bs; 25.4 kg) is converted to approximately 17 lb (7.7 kg) of DDGS, nearly 18 lb (8.2 kg) of ethanol, along with a similar quantity of carbon dioxide (Jaques et al., 2003). Most fuel ethanol production plants are located in the Midwest region of the US, as this area is the locus of the Corn Belt (NASS, 2007). DDGS consists of non-fermented starch and sugars, as well as non-fermentable proteins, fibers, lipids, and minerals. It typically contains roughly 86–93% (db) dry matter, 26–34% (db) crude protein, and 3–13% (db) crude fat (Rosentrater and Muthukumarappan, 2006). Because DDGS is highly nutritious, it is primarily utilized as a livestock feed ingredient.

Due to the dramatic increase in DDGS production that has accompanied the expansion of the ethanol industry during the past decade, these materials are increasingly being transported greater distances via truck and rail and are being stored in various structures, such as bins and silos, until final use at livestock production facilities. But discharge flow is often problematic due to caking and bridging between particles; this behavior frequently occurs during storage and transport. In fact, flowability has become one of the major challenges that needs to be addressed for effective sales, marketing, distribution, and utilization of distillers grains. For example, because these coproducts do not easily flow from rail cars, in order to induce flow workers often hammer the car sides and hopper bottoms. This can lead to severe damage to the rail cars, repairs of which are very expensive. In fact, large rail carriers, such as the BNSF and UP railroads have even prohibited DDGS shipment on their own cars–which has led to increased costs for ethanol plants and feed processors.

Flowability problems in DDGS may arise from the synergistic effects of environmental factors (such as ambient humidity and temperature), compositional properties (including moisture content, fat levels, molecular conformations, and chemical bonding), inherent material properties (i.e., particle size, roughness, shape), as well as storage time, compaction, pressure distribution throughout the product mass, and/or variations in the levels of above listed factors (Craik and Miller, 1958; Fitzpatrick et al., 2004a; Fitzpatrick et al., 2004b; Johanson, 1978; Moreyra and Peleg, 1981; Teunou et al., 1999).

However, there is currently an incomplete understanding about how these individual factors influence flowability, let alone the effects of several factors simultaneously. Although there is some anecdotal knowledge about DDGS flowability from various sources in the industry, this is often propriety in nature and is not comprehensive (Rosentrater and Giglio, 2005; Rosentrater 2006b; Rosentrater 2006c). In terms of scientific studies on DDGS flowability, only a handful of investigations have been published to date. Ganesan et al. (2008a) examined the effects of moisture content and soluble level on the flow behavior of DDGS, and found that dispersibility, flowability index, and floodability index worsened as moisture and soluble levels increased. Ganesan et al. (2008b) again found that increased moisture and soluble levels resulted in decreased flowability; but the addition of a flow agent (CaCO_3_) did not help. In another study (Ganesan et al., 2007a), DDGS flow property data obtained from conventional Carr (1965) and Jenike (1964) testing were numerically modeled using exploratory data analysis techniques, dimensional analysis and response surface methods.

Almost all published research either examines DDGS that has been produced commercially, or within a laboratory setting, and generally does not consider the impacts of processing conditions upon the resulting DDGS. Probst et al. (2013) and Kingsly et al. (2010) examined the impact of adding condensed distillers solubles and drying operations on the resulting chemical and physical properties of DDGS. But neither were comprehensive vis-à-vis flowability properties nor both upsteam as well as downstream plant operations. Thus, the objective of this research was to conduct physical and flowability property analyses on DDGS samples which were produced under varying pilot-scale manufacturing conditions, in order to investigate the effects of various operations (specifically ethanol production and drying conditions) on the resulting properties of the DDGS.

## Materials and Methods

### Pilot Scale Ethanol Manufacturing

Pilot plant trials (*n* = 138 runs) were conducted at the National Corn to Ethanol Research Center, Southern Illinois University, in Edwardsville, IL. A series of experimental runs were conducted that examined the effects of altering front end processing conditions and back end drying conditions (Table 1). The experiments that focused on front end variables were primarily concerned with how liquefaction, saccharification, and solids processing conditions (which influence fermentation performance) impacted the resulting DDGS. The trials that examined drying conditions were primarily concerned with the flow rates of CDS, DWG (i.e., wet cake), and dryer temperature. During the experiments, several processing variables were held constant, including corn feed rate of 500 l b/h, process water temperature of 200°F, process water density of 0.96 g/cm^3^, jet cooker flow rate of 3 gal/min (11.35 L), second and third mash tank levels of 75%, fermenter volume of 3,600 gal (13,627.5 L), fermenter temperature of 90°F (32.2°C), and corn solids density of 1.47 g/cm^3^. An in-depth discussion of the experimental design and implementation of the trials has been provided in Wrenn (2009).

**TABLE 1:** Summary statistics for each independent variable used in the study.^a^

	Coded variable	Actual variable	N	Mean	SD	Minimum	Maximum
Ethanol production front-end variables	FE1	Hammermill screen size (in)	138	0.09	0.02	0.06	0.11
	FE2	Process water flow (lb/min)	138	16.49	1.38	15.01	18.12
	FE3	Solids concentration (%)	138	0.30	0.02	0.28	0.32
	FE4	Slurry density (lb/gal)	138	9.30	0.07	9.21	9.38
	FE5	Slurry tank temperature (F)	138	188.88	5.83	181.52	195.46
	FE6	Slurry tank level (%)	138	76.13	13.98	59.93	90.01
	FE7	Slurry residence time, tank 1 (min)	138	25.38	4.66	19.98	30.00
	FE8	Slurry residence time, tank 2 (min)	138	28.43	5.62	20.78	35.90
	FE9	Slurry pH	138	5.80	0.18	5.45	6.17
	FE10	Slurry tank enzyme flow (g/h)	138	83.00	23.01	0.00	103.06
	FE11	Jet cooker temperature (F)	138	229.58	4.68	224.91	235.04
	FE12	Mash tank temperature (F)	138	186.10	4.12	180.72	192.51
	FE13	Liquefaction mash tank level (%)	138	74.68	9.36	64.54	85.10
	FE14	Liquefaction mash residence time, tank 1 (min) 138	124.46	15.60	107.56	141.82	
	FE15	Liquefaction mash residence time, tank 2 (min) 138	139.27	20.09	111.85	168.99	
	FE16	Liquefaction mash tank enzyme flow (gal/h)	138	165.82	45.37	0.00	207.44
Ethanol production back-end variables	BE1	Syrup addition rate (dry kg/h)	138	11.90	3.60	0.00	18.38
	BE2	Mass flow rate of wet DDGS (wet kg/h)	138	53.77	16.47	0.00	96.37
	BE3	Mass flow rate of dry DDGS (dry kg/h)	138	46.46	14.19	0.00	84.28
	BE4	Dryer temperature (F)	138	222.18	13.27	152.97	243.42
	BE5	Mass flow rate of w etcake (wet kg/h)	138	91.81	32.00	0.00	166.00
	BE6	Mass flow rate of w etcake (dry kg/h)	138	31.49	10.97	0.00	56.94

a FE denotes front end processing; BE denotes back end processing; specific process conditions are fully explained in Wrenn (2009).

### Analysis of DDGS

After production, DDGS samples were stored under refrigerated conditions (4 ± 1°C) in sealed plastic containers. When needed for experimentation (∼a few months time), the samples were equilibrated to room temperature (24 ± 1°C). Extensive physical and flowability analyses were conducted on the DDGS samples following methods described by Ganesan et al. (2008c). These included moisture content, water activity, thermal properties, flowability properties, and color. All property determinations, except moisture content, were conducted at room temperature. All physical and flowability properties were determined using three replicates (*n* = 3) for each sample; each property was studied using a completely randomized design.

Moisture content was determined following RFA recommendations (www.ethanolrfa.org), using a forced-convection laboratory oven (Thelco Precision, Jovan Inc., Wincester, VA) at 105°C for 3 h. Water activity was measured using a calibrated water activity meter (AW Sprint TH 500, Novasina, Talstrasse, Switzerland). Thermal conductivity, resistivity, and diffusivity were determined with a thermal properties meter (KD2, Decagon Devices, Pullman, WA), that utilized the line heat-source probe technique (Baghe-Khandan et al., 1981). Flowability properties included angle of repose, aerated (i.e., loose) bulk density, packed bulk density, Hausner Ratio, compressibility, angle of spatula, uniformity, total flowability index, angle of fall, angle of difference, dispersibility, flow index, and total floodability index. These were determined through Carr index testing (Carr, 1965) using a powder characteristics tester (Model PTR, Hosokawa Micron Powder systems, Summit, NJ) following the procedures described by ASTM (1999). Additionally, the geometric mean diameter and geometric standard deviation of the particles were calculated following ASAE (2004) using a Rotap sieve analyzer (Mentor, OH). Color was measured using a spectrophotocolorimeter (LabScan XE, Hunter Associates Laboratory, Reston, VA) using the L-a-b opposable color scales (Hunter Associates Laboratory, 2002), as well as the 457 Brightness (i.e., whiteness), Gardner 10 and Gardner 20 (i.e., yellowness) indices.

Summary statistics were determined using Microsoft Excel v.2003 (Microsoft Corp., Redmond, WA). Pearson product-moment linear correlations were quantified among all measured variables using SAS v.8 (SAS Institute, Cary, NC). Scatterplot matrices, linear regressions, and multivariate statistics (Principal Components Analysis (PCA), and Partial Least Squares (PLS) regression) were constructed using Minitab v. 14.11 (Minitab Inc., State College, PA).

## Results and Discussion

### Univariate Analysis

Univariate summary statistics for both chemical properties as well as physical and flowability properties are provided in Table 2. All chemical constituents had fairly broad ranges, as shown in Table 2 (e.g., protein had a standard deviation of 5.22; NDF had 4.78; solids had 4.35), which was the result of either front end processing conditions, back end conditions, or combinations thereof. Wrenn (2009) provides an in-depth discussion about the specific effects that the experimental manufacturing conditions had on the DDGS chemical properties.

**TABLE 2:** Summary statistics for each dependent variable measured in the study.^a^

	Coded variable	Actual variable	N	Mean	SD	Minimum	Maximum
DDGS composition	Protein	Crude protein (% db)	138	30.05	5.22	12.77	42.25
	Fat	Crude fat (% db)	138	12.63	2.46	5.15	17.62
	NDF	Neutral detergent fiber (% db)	138	29.73	4.78	19.59	46.87
	ADF	Acid detergent fiber (% db)	138	15.43	2.59	9.71	22.95
	Ash	Ash (% db)	138	4.33	0.85	2.14	6.38
	Solids	Dry solids (%)	138	85.71	4.35	67.89	96.44
DDGS physical and flow properties	MC	Moisture content (% db)	363	16.26	4.68	4.71	32.47
	AW	Water activity (-)	363	0.55	0.10	0.15	0.87
	Thermalcond	Thermal conductivity (W/m C)	363	0.07	0.01	0.06	0.08
	Thermalresis	Thermal resistivity (m C/W)	363	13.65	0.85	11.80	16.80
	Thermaldiff	Thermal diffusivity (mm^2^/s)	363	0.13	0.01	0.12	0.16
	AoR	Angle of Repose (deg)	363	46.14	2.71	38.00	54.50
	ABD	Aerated bulk density (g/cm^3^)	363	0.60	0.06	0.39	0.73
	PBD	Packed bulk density (g/cm^3^)	363	0.63	0.06	0.43	0.75
	HR	Hausner ratio (−)	363	1.06	0.02	1.00	1.13
	Compressibility	Compressibility (%)	363	5.26	1.58	0.08	11.30
	AoS	Angle of spatula (deg)	363	58.32	3.61	46.70	66.35
	Uniformity	Uniformity (−)	363	7.51	3.48	0.10	20.80
	Totalflow index	Total flow ability index (−)	363	73.62	3.76	59.00	87.50
	AoF	Angle of fall (deg)	363	41.15	3.24	26.50	51.70
	AoD	Angle of difference (deg)	363	4.99	3.10	0.10	21.50
	Dispers	Dispersibility (%)	363	17.50	7.90	6.60	64.20
	Flow index	Flow ability index (−)	363	24.98	0.37	18.00	25.00
	Totalfloodindex	Total floodability index (−)	363	56.97	5.08	46.00	77.00
	L	Hunter L value	363	40.35	3.98	28.79	53.15
	a	Hunter a value	363	11.52	1.49	5.79	14.19
	b	Hunter b value	363	20.99	1.90	12.66	25.13
	457Bright	457 brightness index	363	3.71	1.42	2.07	14.15
	Gardner10	Gardner 10 yellow index	363	10.15	0.63	5.80	11.00
	Gardner20	Gardner 20 yellow index	363	8.51	0.59	4.40	9.30

a Summary statistics consider all samples; the effects of process variables on these properties are described in subsequent tables.

The majority of the physical and flow properties also had broad ranges. In fact, most variables exhibited maximum values which were at least twice as large (or even greater) than the minimum values. For example, moisture content ranged from 4.71 to 32.47% (db), while water activity ranged from 0.150 to 0.870. Additionally, most properties exhibited a fair amount of variability, but with some more than others. Examining all of the properties in Table 2, it appears that some of the DDGS samples had mean values very similar to those which are found commercially (Bhadra et al., 2009; Rosentrater, 2006a; ASABE, 2020), but it also appears that many of the DDGS samples had properties which were either higher or lower than typical commercial samples; this is shown by their broad ranges. Examples of this include moisture content, water activity, and most flowability properties. For all samples studied, however, thermal properties (which had a very narrow range) and color values (which had a very broad range) were very reflective of the ranges typically found in commercial samples. Examining univariate statistics is useful, but more information can be found through bivariate and multivariate analyses, both of which examine the potential effects due to the independent variables and interactions among them.

### Bivariate Analysis

**FIGURE 1:**
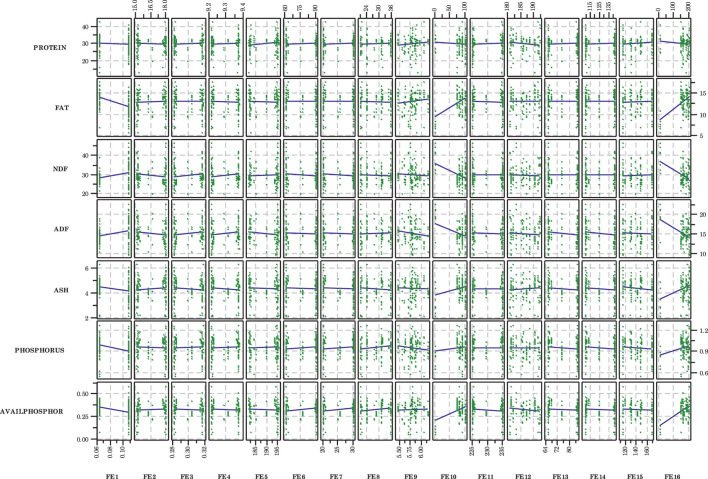
Scatterplots of compositional variables for each front end independent variable. Although considerable scatter is present, some linear trends appear pronounced. Variable definitions provided in Tables 1, 2.

**FIGURE 2:**
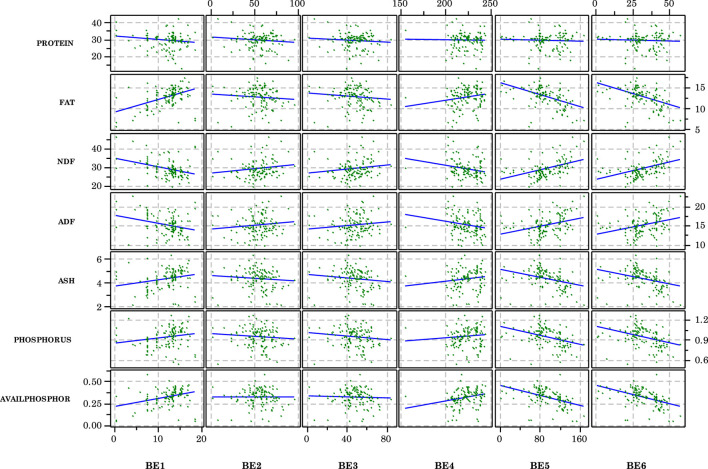
Scatterplots of compositional variables for each back end independent variable. Although considerable scatter is present, some linear trends appear pronounced. Variable definitions provided in Tables 1, 2.

The effects of the front end processing variables on DDGS composition is shown in Figure 1; the effects of the back end variables are shown in Figure 2. In terms of front end variables, FE1 (hammermill screen size), FE10 (slurry tank enzyme flow rate), and FE 16 (liquefaction mash tank enzyme flow rate) appeared to have the greatest influences on resulting DDGS chemical composition. Back end conditions also appeared to influence DDGS composition, especially BE1 (syrup addition rate), BE5 [mass flow rate of wet cake (dry)], and BE6 [mass flow rate of wet cake (wet)]. It should be noted that considerable variability was present in the collected data, which made definitive relationships difficult to determine. A more detailed discussion about the effects of the processing conditions on DDGS composition is provided in Wrenn (2009).

**FIGURE 3:**
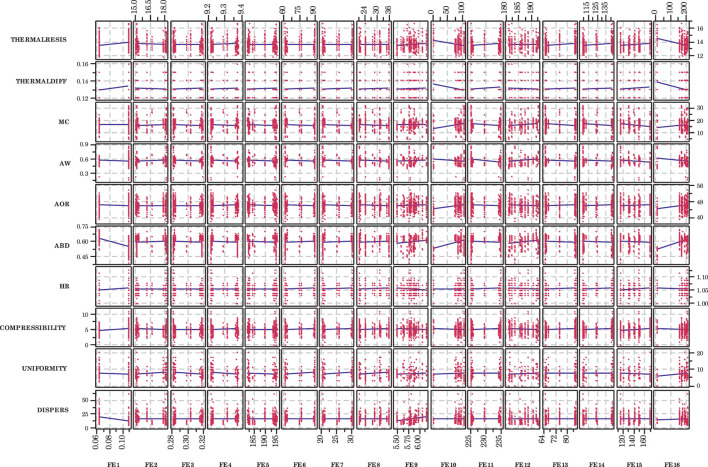
Scatterplots of dependent variables for each front end independent variable. Although considerable scatter is present, some linear trends appear pronounced. Variable definitions provided in Tables 1, 2.

Physical and flowability properties of the DDGS appeared to be mostly unaffected by the front end independent variables (Figure 3). In fact, water activity, Hausner Ratio, compressibility, and uniformity appeared to exhibit almost no change as the value of each independent variable increased. The rest of the dependent variables had similar behavior. A few of the exceptions included hammermill screen size (FE1), which seemed to affect the aerated bulk density (ABD) and dispersibility; slurry pH (FE9), which appeared to affect dispersibility; slurry tank enzyme flow rate (FE10), which appeared to affect thermal resistivity, thermal diffusivity, moisture content, angle of repose, and aerated bulk density; and liquefaction mash tank enzyme flow rate (FE16), which appeared to impact thermal diffusivity, angle of repose, and aerated bulk density. There was considerable scatter in the data, which made definitive trends difficult to discern. It is true that scatter diminishes the usability of data, but it must be understood that biological processes inherently and often exhibit considerably higher variability. Even so, thorough statistical analyses can often yield meaningful and usable trends.

**FIGURE 4:**
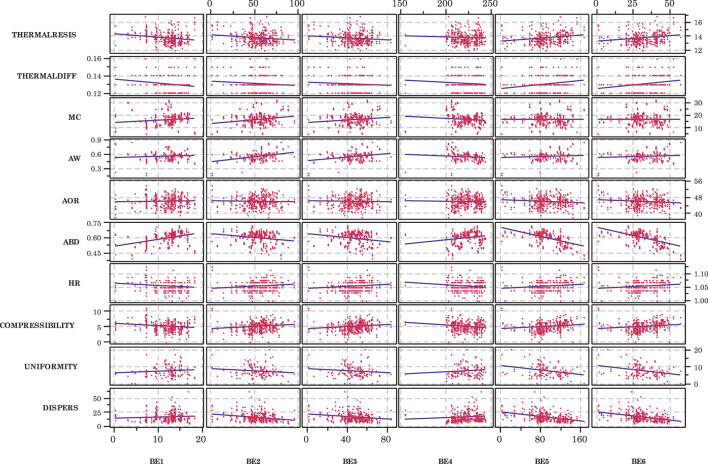
Scatterplots of dependent variables for each back end independent variable. Although considerable scatter is present, some linear trends appear pronounced. Variable definitions provided in Tables 1, 2.

The back end independent variables, on the other hand, appeared to have marginally greater influences on the DDGS physical and flowability properties (Figure 4), although some appeared to be more influential than others. For example, it appeared that angle of repose was linearly related to all of the back end variables; particle uniformity appeared to decrease as wet cake mass flow rate (wet) increased (BE5) and as wet cake mass flow rate (dry) increased (BE6); dispersibility also appeared to decrease as BE5 and BE6 increased. Other linear trends were not as clear, however. Again, considerable scatter in the data made it difficult to have certainty in these potential relationships.

It is also important to examine potential relationships between the dependent variables, especially in terms of identifying and quantifying factors which influence DDGS flowability. Potential linear relationships among all dependent variables were investigated using linear correlation analysis. Table 3 lists those correlations which were significant (*α* = 0.05) and were greater than |0.70|. Table 4 summarizes linear regressions for these relationships, which were constructed as specific flowability variables as a function of either a compositional variable or another physical or flowability variable. It appeared that both aerated and pack bulk densities were influenced by DDGS fat content (as evidenced by their high correlation (Table 3) and coefficient of determination (Table 4) values). Anecdotally, flowability has been seen to decrease as fat content increases; these results seem to support that hypothesis, and in fact may be the single most important findings of this study. Color indices (457 Brightness, Gardner10, and Gardner 20) were impacted by the DDGS fat content as well. This was probably because the fat in DDGS contains carotenoid pigments. Both aerated and packed bulk densities were influenced by fiber (specifically NDF) content, which is not surprising, because flowability of granular materials is impacted by particle size and shape. In DDGS, particles high in fiber generally have unique structures compared to those which are high in protein and fat. Not surprisingly, thermal properties were related to each other, primarily because the definition of thermal diffusivity encompasses thermal conductivity (in addition to mass density and specific heat…which thus encapsulates ability to conduct heat vis-à-vis store heat energy). The thermal properties of the DDGS in this study are quite similar to those of other grain-based meals. Additionally, both aerated and packed bulk densities appeared to be linearly related to various color variables. Perhaps color is a surrogate variable that actually is indicative of processing conditions or other DDGS properties. Finally, Hausner Ratio was found to be linearly related to compressibility. This was not surprising either, because both of these are defined in terms of each of the bulk densities.

**TABLE 3:** Significant (*p* < 0.05) Pearson linear correlations between dependent variables (which are impacted by front end and back end processing conditions).

Variable associations			Correlation coefficient (r)
Fat	×	ABD	0.7039
Fat	×	PBD	0.7148
Fat	×	457 brightness index	−0.7087
Fat	×	Gardner 10 yellow index	0.7191
Fat	×	Gardner 20 yellow index	0.7145
NDF	×	ABD	-0.8215
NDF	×	PBD	−0.8229
NDF	×	L	0.7209
NDF	×	457 brightness index	0.7107
Thermal conductivity	×	Thermal resistivity	−0.8514
Thermal conductivity	×	Thermal diffusivity	−0.7636
Thermal resistivity	×	Thermal diffusivity	0.9034
ABD	×	PBD	0.9887
ABD	×	L	−0.7795
PBD	×	L	−0.7741
PBD	×	457 brightness index	−0.7505
PBD	×	Gardner 10 yellow index	0.7141
PBD	×	Gardner 20 yellow index	0.7114
HR	×	Compressibility	0.9646

**TABLE 4:** Linear regression results for those variable combinations which exhibited significant linear correlations.

	Prediction equation					—		
Response	Intercept	+	Predictor variable	×	Coefficient	MS regression	MS error	R^2^
ABD	0.358		Fat		0.019	0.696	0.002	0.488
PBD	0.390		Fat		0.019	0.700	0.002	0.510
457 Brightness	9.240		Fat		−0.428	364.680	1.000	0.501
Gardner 10	7.650		Fat		0.193	74.379	0.192	0.516
Gardner 20	6.170		Fat		0.181	65.246	0.173	0.509
ABD	0.909		NDF		−0.011	0.951	0.001	0.668
PBD	0.938		NDF		−0.010	0.927	0.001	0.677
L	23.000		NDF		0.589	2986.500	7.600	0.518
457 Brightness	−2.370		NDF		0.206	366.690	1.000	0.504
Thermal conductivity	0.152		Thermal resistance		−0.006	0.009	0.001	0.724
Thermal diffusivity	0.211		Thermal conductivity		−1.090	0.014	0.001	0.582
Thermal diffusivity	0.012		Thermal resistance		0.009	0.020	0.001	0.816
PBD	0.052		ABD		0.968	1.332	0.001	0.974
ABD	1.090		L		−0.012	0.857	0.002	0.602
PBD	1.110		L		0.012	0.819	0.002	0.598
PBD	0.753		457 Brightness		−0.033	0.768	0.002	0.561
PBD	−0.073		Gardner 10		—	0.694	0.002	0.506
PBD	0.008		Gardner 20		0.073	0.689	0.002	0.502
HR	0.997		Compressibility		0.011	0.113	0.001	0.905

**FIGURE 5:**
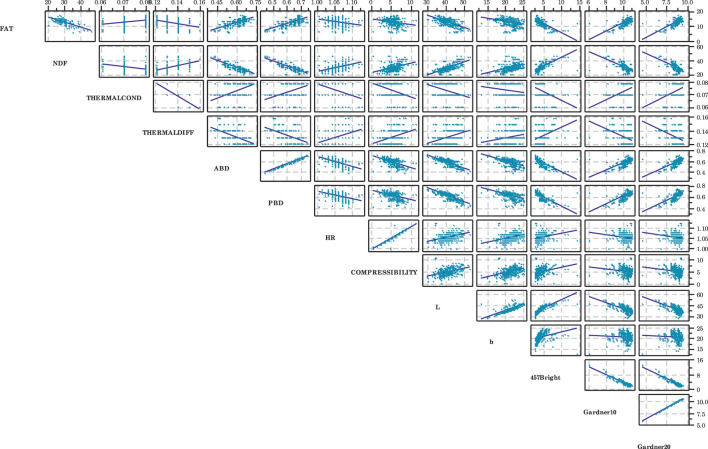
Scatterplot matrix for those variables which exhibited significant linear correlations. Many variables appear to have strong relationships. Variable definitions provided in Tables 1, 2.

Figure 5 provides a scatterplot matrix of all bivariate combinations of the dependent variables. This approach provides more information than just the correlation or linear regression analyses can provide, because it depicts all potential relationships simultaneously. Most of the plots exhibited fairly high variability. Several of the plots indicate outlying data points, which may influence the resulting relationships (i.e., Hausner Ratio vs. Gardner 10; compressibility vs. Garner 10). But some of the outliers appear to have little influence (i.e., Hausner Ratio vs. compressibility). A few plots exhibited extremely low scatter (i.e., aerated bulk density vs. packed bulk density; Gardner 10 vs. Gardner 20).

### Multivariate Analysis

**FIGURE 6:**
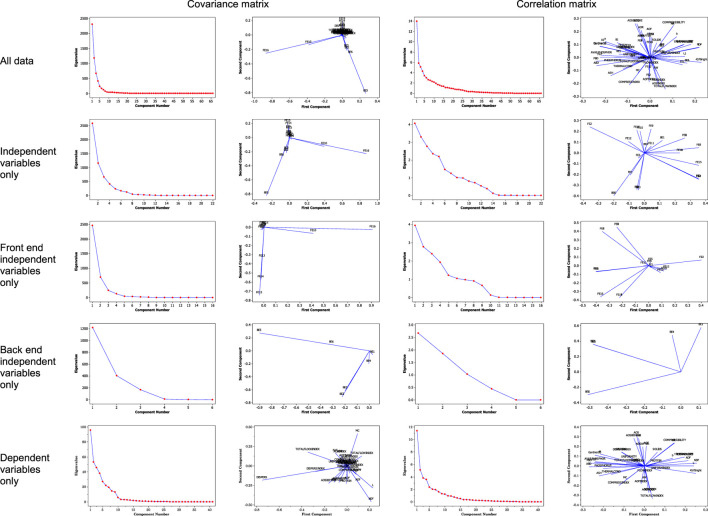
Error and loading plots for Principal Components Analysis (PCA).

Multivariate analyses consisted of Principal Components Analysis (PCA) and Partial Least Squares (PLS) regression. PCA results (Figure 6) provided some insight into the multivariate structure of the dataset. One of the most important features of PCA is that it provides a summary of the multivariate data using a reduced dimensionality compared to all of the original variables which are present in the data. In that regard, PCA was successful on all accounts. But, as shown in the figure, the choice of using either the covariance or the correlation matrix to quantify variation in the data did affect the resulting performance of the PCA. For example, PCA encompassing all of independent and dependent variables simultaneously with the covariance matrix indicated that at least five components were required to summarize the data (shown in the error plot), while two variables [liquefaction mash tank enzyme flow (FE16) and mass flowrate of wet cake (BE5)] were highly influential (indicated by the loading plot) on the data. Using the correlation matrix, on the other hand, either two or five components were required to summarize all of the data (as observed in the error plot), but most of the variables were accounted for (seen in the loading plot) – which means that the correlation matrix was better at summarizing the data compared to the covariance. Examining PCA results using only the independent variables with the covariance matrix, it appears that either three or eight components would be required, while two variables [liquefaction mash tank enzyme flow (FE16) and mass flowrate of wet cake (BE5)] were highly influential; PCA using the correlation matrix, on the other hand, indicated that either six or 14 components would be needed, although most of the variables are accounted for in the model. Conducting PCA using only the front end independent variables, it appears that with the covariance matrix, either three or five components are required, and several variables were highly influential (FE10, FE13, FE14, FE15, and FE16 – see Table 1 for variable definitions) on the multivariate data; results using the correlation matrix were less clear, however; either five or 10 components were required to summarize the data, and nearly half of the front end variables were deemed influential. PCA using only the back end independent variables with the covariance matrix resulted in either two or four components needed, and all variables except BE1 (syrup addition rate) and BE4 (dryer temperature) showed a high level of influence; results using the correlation matrix, on the other hand, indicated that five components were required to summarize the data, and all of the back end variables were influential. PCA using only the dependent variables (all composition, physical, and flowability properties) with the covariance matrix indicated that either five or 10 components were required to summarize the multivariate data, and while many of the dependent variables were influential, only dispersibility had an extremely high effect on the multivariate data structure; PCA with the correlation matrix indicated that five components were needed, and almost all of the dependent variables were accounted for. Overall, PCA using the correlation matrix appeared to provide somewhat better summaries of the multivariate data than those with the covariance matrix. PCA is an effective mechanism for summarizing and examining multivariate data, but it did not provide high predictive capability. This may be achieved via PLS regression.

**FIGURE 7:**
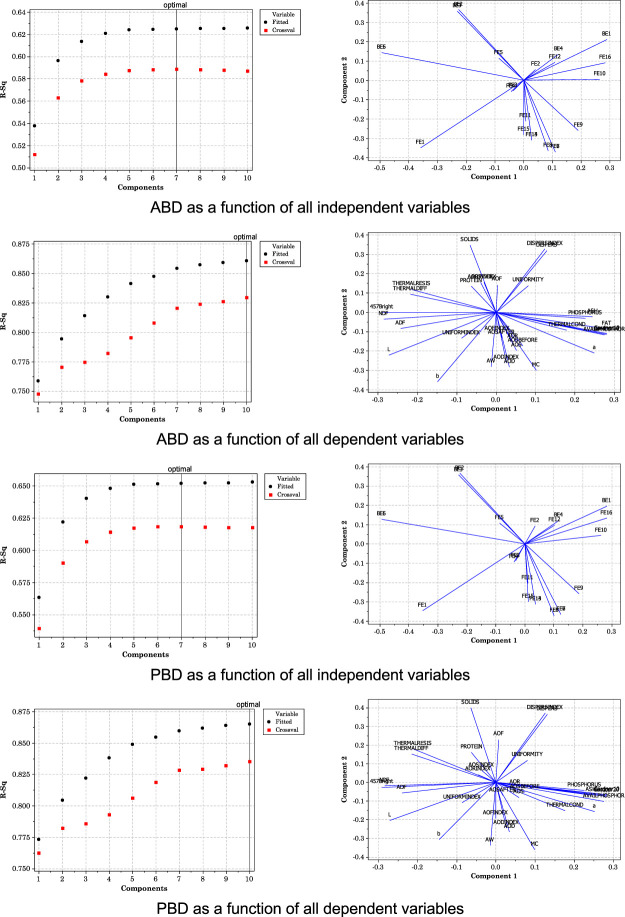
Model selection and loading plots for ABD and PBD from PLS regression.

Because aerated bulk density and packed bulk density are two key flowability parameters in granular materials such as DDGS (Bhadra et al., 2009), these were selected for PLS regression analysis. PLS did provide fairly adequate descriptions of the multivariate data relative to both of these flowability variables (Figure 7). Aerated bulk density could be modeled as a function of all the independent variables with a resulting R^2^ value greater than 62% (seven components were required), and as a function of all dependent variables with an R^2^ greater than 85% (using 10 components). Packed bulk density could be modeled as a function of all independent variables with a resulting R^2^ greater than 65% (seven components were required), and as a function of all dependent variables with an R^2^ greater than 86% (using 10 components). For all of the PLS regressions, most of the independent and dependent variables were highly influential on the multivariate data structure, as indicated by the respective loading plots. Should PCA and PLS regression be used to more thoroughly predict flowability behavior, or will better performance be achieved measuring fat content alone?

## Conclusion

A series of pilot-scale ethanol production trials were conducted to examine the effects of front end as well as back end processing conditions on DDGS physical and flowability properties. There was considerable scatter in the data, and thus it was difficult to discern clear patterns or relationships among the independent and dependent variables. Principal Components Analysis and Partial Least Squares Regression were only marginally successful in summarizing the multivariate data. Even so, several insights were gained. DDGS color (Hunter L, a, b) values were influenced by processing conditions; they were also highly correlated with fat content, and with aerated and packed bulk densities. It appears that color may be a surrogate for other variables which were not actually measured in this study, but have an influence on physical and flow properties. The most important finding from this study, however, was that DDGS fat content was highly correlated with both aerated and packed bulk densities; thus it appears that fat level plays a key role in flowability behavior. Future studies should examine this potential relationship in more depth, especially as the industry has moved to fat reduction via oil separation processes.

## Data Availability

The raw data supporting the conclusion of this article will be made available by the authors, without undue reservation.
